# Acromegaly Type 1: A Representative Patient

**DOI:** 10.1210/jcemcr/luae053

**Published:** 2024-04-17

**Authors:** Daniel Cuevas-Ramos, Shlomo Melmed

**Affiliations:** Neuroendocrinology Clinic, Department of Endocrinology and Metabolism, Instituto Nacional de Ciencias Médicas y Nutrición Salvador Zubirán, 14080 Mexico City, Mexico; Pituitary Center, Department of Medicine, Cedars-Sinai Medical Center, Los Angeles, CA 90048, USA

**Keywords:** growth hormone, insulin-like growth factor type 1, pituitary adenoma, neuroendocrine tumor, IGF-1

## Abstract

A 46-year-old woman was troubled by a 3-year history of constant headaches and arthralgias. She was treated with paracetamol with no symptom resolution. An abnormal fasting glucose level prompted endocrine evaluation. On physical examination, she casually mentioned that her wedding ring no longer fit, and she also confirmed an increase in shoe size. There were no characteristic facial features for acromegaly and there was no evidence of acral enlargement. Biochemical evaluation, including insulin-like growth factor type 1 (IGF-1) measurement and oral glucose loading with growth hormone (GH) measurement confirmed excess GH production and a diagnosis of acromegaly. Pituitary magnetic resonance imaging showed a central pituitary microadenoma. After transsphenoidal surgical resection, tissue immunohistochemistry revealed a densely granulated somatotroph adenoma. Currently, the patient is asymptomatic with biochemical disease control, normal fasting glucose levels, and no pituitary hormone deficiencies. This patient is illustrative of a type 1 acromegaly with mild clinical manifestations. Clinicians should be aware of acromegaly subtypes to avoid delay in diagnosis and to individualize therapy.

## Introduction

Acromegaly is a chronic disorder causing increased mortality if untreated (1). The most common cause is a growth hormone (GH)-secreting pituitary adenoma (2, 3). Traditionally, acromegaly is considered to present with clear clinical features, including characteristic facial and acral changes, which trigger diagnosis. In reality, somatotroph adenomas may exhibit variability in clinical presentation, related to structural and functional differences in the tumor. We have classified 3 different clinical phenotypes of this disease and their respective outcomes (4). Here we illustrate a type 1 acromegaly patient, characterized by very mild manifestations of the disorder. Clinicians should be aware of distinct acromegaly phenotypes to avoid a delay in diagnosis and appropriate therapy.

## Case Presentation

**Figure 1.**
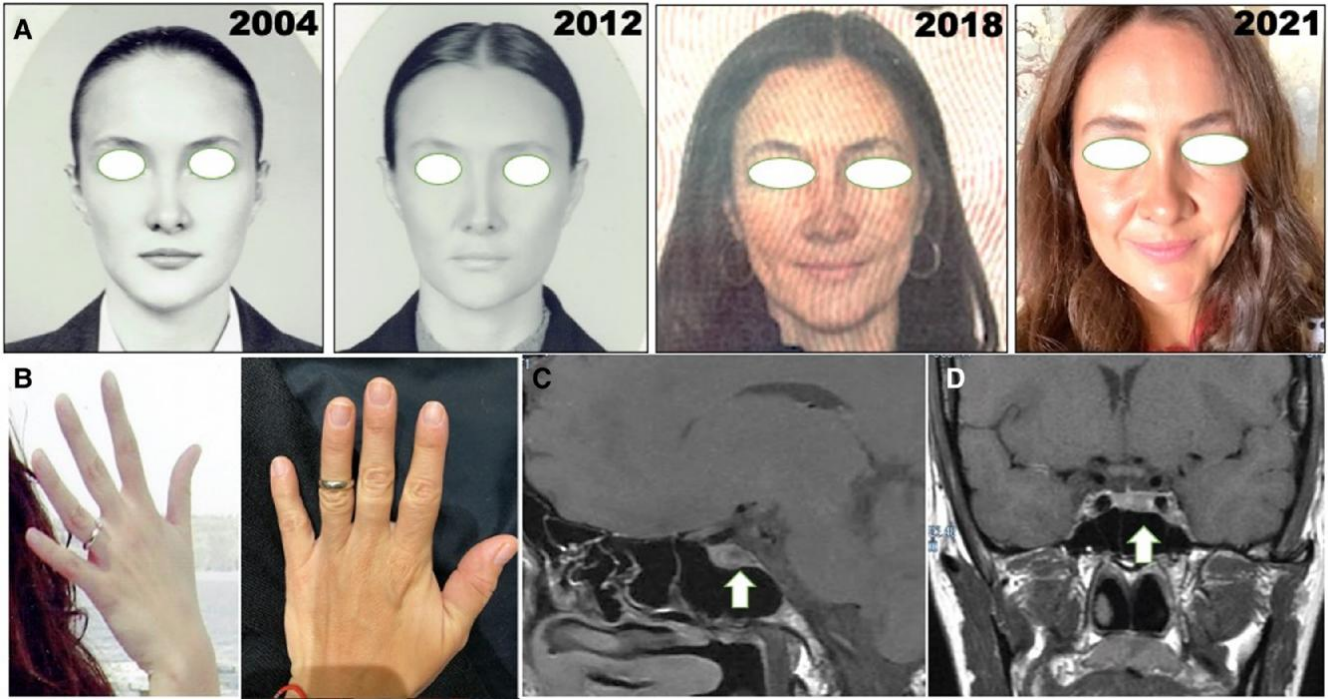
A. Mild clinical changes in the patient over time with prognathism, frontal bone bossing, a wider nose, and increased face skin folds apparent in photographs. B. Failure to fit the wedding ring at diagnosis in comparison to past. C. Microadenoma identified with T1 gadolinium-enhanced MRI sequence in the middle/center of the pituitary (white arrow). D. Coronal T1 gadolinium-enhanced MRI sequence showing the microadenoma (white arrow).

A 46-year-old woman presented with a 3-year history of chronic headache and arthralgias. She had no obvious facial or acral features consistent for a clinical suspicion of acromegaly (Fig. 1A). Despite numerous biochemical evaluations for chronic diseases, migraine, neuropathies, and autoimmune and inflammatory markers, there was no definite diagnosis. She was only treated with anxiolytics and antidepressants. She developed an abnormal fasting plasma glucose level of 106 mg/dL, but did not have obesity or other risk factors. Subsequent endocrine evaluation revealed arthralgias in both hands, without arthritis. On physical examination for synovitis, she reported that her wedding ring no longer fit (Fig. 1B) and also confirmed an increase in shoe size. Further physical examination was normal.

## Diagnostic Assessment

The patient reported a history compatible with acral enlargement, including change in ring and shoe sizes (Fig. 1B). A 75-g glucose tolerance test (OGTT) revealed a nonsuppressed GH level and slightly elevated serum insulin-like growth factor type 1 (IGF-1) (Table 1). Pituitary magnetic resonance imaging with a gadolinium-enhanced T1 sequence revealed a central hypointense microadenoma of 9 × 5 × 7 mm (Fig. 1C and 1D, white arrows). Type 1 acromegaly was suspected based on the mild acromegaly symptomatology, discrete acral changes, low disease activity, and the presence of a microadenoma (4).

**Table 1.** Biochemical and histologic results

	Results		Reference values
***At diagnosis***			
IGF-1	430 ng/mL (56.2 nmol/L)		71–253 ng/mL (9–33 nmol/L)
PRL	44 ng/mL (44 mcg/L)		<26 ng/mL (mcg/L)
*OGTT*	*Glucose*	*GH*	
0 minutes	94 mg/dL (5.2 mmol/L)	3.2 ng/mL (3.2 ng/mL)	<5 ng/mL (<5 ng/mL)
30 minutes	97 mg/dL (5.3 mmol/L)	2.6 ng/mL (2.6 ng/mL)	<0.4 ng/mL (<0.4 ng/mL)
60 minutes	89 mg/dL (4.9 mmol/L)	2.8 ng/mL (2.8 ng/mL)	<0.4 ng/mL (<0.4 ng/mL)
90 minutes	109 mg/dL (6.0 mmol/L)	2.4 ng/mL (2.4 ng/mL)	<0.4 ng/mL (<0.4 ng/mL)
120 minutes	135 mg/dL (7.5 mmol/L)	2.9 ng/mL (2.9 ng/mL)	<0.4 ng/mL (<0.4 ng/mL)
180 minutes	84 mg/dL (4.6 mmol/L)	3.5 ng/mL (3.5 ng/mL)	<0.4 ng/mL (<0.4 ng/mL)
240 minutes	79 mg/dL (4.4 mmol/L)	8.7 ng/mL (8.7 ng/mL)	<0.4 ng/mL (<0.4 ng/mL)
300 minutes	70 mg/dL (3.9 mmol/L)	4.4 ng/mL (4.4 ng/mL)	<0.4 ng/mL (<0.4 ng/mL)
***After surgery***			
GH	0.38 ng/mL (0.38 ng/mL)		<5 ng/mL (<5 ng/mL)
IGF-1 ng/mL (nmol/L)			
After 1 month	215 ng/mL (28 nmol/L)		71–253 ng/mL (9–33 nmol/L)
After 3 months	124 ng/mL (16 nmol/L)		71–253 ng/mL (9–33 nmol/L)
After 1 year	112 ng/mL (14.6 nmol/L)		71–253 ng/mL (9–33 nmol/L)
PRL ng/mL (or mcg/L)	52 ng/mL (52 ng/mL)		<26 ng/mL (<26 ng/mL)
***Adenoma immunochemistry***			
Chromogranin	Positive ++		
GH staining	Positive ++		
PRL staining	Negative		
Granulation	Densely granulated		
p21	Positive +++		
SSTR2	Positive +++		

Abbreviations: GH, growth hormone; IGF, insulin growth factor; OGTT, 75-g oral glucose tolerance test; PRL, prolactin; SSTR2, type 2 somatostatin receptor.

## Treatment

Transsphenoidal surgical resection yielded a densely granulated somatotroph pituitary adenoma which was strongly positive for GH, protein 21 (p21), and somatostatin type 2 receptor (SSTR2) (Table 1).

## Outcome and Follow-Up

After 3 months follow-up, pituitary hormone levels were normal; serum fasting glucose and IGF-1 also normalized (Table 1). A magnetic resonance study showed no evidence of a residual pituitary lesion. Retrospective review of patient photographs (Fig. 1A), showed subtle progressive jaw prognathism and frontal bone bossing; no florid acromegaly facial changes were noted. After 1 year the patient remains in remission, with normal pituitary function.

## Discussion

Acromegaly is a chronic disease, marked by indolent progressive clinical complications (2, 3). Prompt therapy is important to mitigate cardiovascular and respiratory morbidity which compromise quality of life and leads to excess mortality (1). The most common cause is a GH-secreting somatotroph adenoma. First-line therapy generally requires neurosurgical resection (5). If disease persists, further medical therapy may be indicated, including somatostatin receptor ligands (SRLs), GH-receptor antagonist, or dopamine agonists. Radiotherapy is also an option (5). However, some patients with acromegaly remain biochemically active despite multiple therapy modalities, resulting in aggressively growing adenomas, higher complication rates, and increased mortality risk (1, 2). In contrast, some patients exhibit a more insidious disease course, with smaller adenomas and lower disease activity. We have evaluated phenotypic differences in acromegaly cases and reported 3 distinct acromegaly categories (Table 2) (4). The type 1 phenotype is characterized by densely granulated somatotroph microadenomas characterized by intense immunochemistry staining of p21 (protein related with cell-cycle arrest and lower cell division), and high SSTR2 expression; these features are all associated with more favorable responsiveness to SRL therapy. Disease activity is less clinically pronounced because most of the densely packed GH granules are not released, as evidenced by immunostaining with cytokeratin 5.2. Consequently, IGF-1 levels are only slightly elevated. As in our case, such patients show the most favorable prognosis with a high probability of disease remission at follow-up and require fewer therapeutic modalities. In contrast, a more severe prognosis tends to manifest in patients with type 3 somatotroph adenomas. This phenotype is characterized by the presence of macroadenomas with less intense p21 staining and, consequently, increased cell division, low SSTR2 (lower responses to SRLs) expression, and the presence of sparse GH granulation on cytokeratin 5.2 immunostaining. Since most GH granules are secreted, IGF-1 levels are particularly high, and disease activity is more pronounced with less probability of remission despite multiple surgical or medical (pharmacological or radiotherapy) interventions. Type 2 macroadenomas show intermediate features between subtypes 1 and 3 (4).

**Table 2.** Acromegaly classification

	Acromegaly		
	Type 1	Type 2	Type 3
**Prevalence**	50%	20%	30%
**Age (years)**	53 ± 12	40 ± 9	32 ± 8
**Years prior to diagnosis**	7–14	5–10	1–4
**Adenoma size**	Microadenoma	Macroadenoma	Macroadenoma
**Cavernous sinus invasion**	Rare	Negative	Common
**Adenoma growth**	Slow	Stable	Aggressive
**Optic chiasm compression**	Never	Rare	Common
**GH granulation**	Densely	Mixed	Sparsely
**SSTR2 expression**	High	Intermediate	Low
**p21 expression**	High	Intermediate	Low
**Prognosis**	Good	Intermediate	Poor

Abbreviations: GH, growth hormone; SSTR2, type 2 somatostatin receptor.

Here we present a case of a type 1 acromegaly patient with mild, nonspecific manifestations (Fig. 1A), who was not correctly diagnosed or adequately treated for at least 3 years. After neurosurgical resection, a densely granulated GH-expressing adenoma was confirmed, with mild biochemical disease activity (Table 1). Since these microadenomas have a greater potential to be completely resected, a more favorable neurosurgical outcome can be expected. If necessary, SRLs are useful to control disease activity because of the high SSTR2 expression (4).

In this case, similar to other type 1 patients, the diagnosis was not suspected; this contrasts with the clear clinical presentation afforded by more aggressive type 2 or 3 adenomas (Table 2) (4). This patient confirms that not all patients present with pathognomonic facies and acral changes characteristic of acromegaly. In patients with chronic headache and arthralgias a diagnosis of acromegaly should be excluded. Of note, the persistent postoperative hyperprolactinemia was likely due to surgical stalk compromise, this situation resolved on follow-up.

Inclusion criteria for clinical trials should consider our subtype classification. Type 1 adenomas respond more favorably to SRLs than type 2 and type 3 adenomas, with normalization of GH and IGF-1 levels. So, if type 1 or 2 adenomas comprise the majority of a trial cohort, higher rates of responsiveness to therapy may be observed (6-8). In contrast, if more aggressive, type 3 adenomas are included (9, 10), trial efficacy outcomes may be expected to be less favorable. In addition, if acromegaly remains well controlled, type 1 patients may be good candidates for extended-dose therapy with SRLs, every 6 to 8 weeks instead of monthly injections (11). In contrast, if type 2 or 3 patients show incomplete responsiveness to SRLs, a high-dose or high-frequency regimen may be necessary to normalize IGF-1 values (12).

## Learning Points

- Structural and functional classification of acromegaly has identified 3 different types of adenomas, with differing disease activity and treatment responsiveness.
- Patients with type 1 acromegaly show milder clinical manifestations, and clinicians should be aware of such patients to avoid a delay in diagnosis and appropriate therapy. Prognosis is favorable, and patients are usually cured with neurosurgery.
- IGF-1 measurement should be included in the differential evaluation of patients presenting with chronic headaches and arthralgias.
- If type 1 microadenomas invade the cavernous sinus, they can be treated first with SRLs to decrease tumor volume, mitigate subsequent invasiveness, and possibly increase probability of complete surgical resection.
- Inclusion criteria for clinical studies should consider acromegaly classification to properly select patients and evaluate treatment responsiveness.

## Data Availability

Data sharing is not applicable to this article as no datasets were generated or analyzed during the current study.

## Abbreviations

GH: growth hormone
IGF-1: insulin-like growth factor type 1
p21: protein 21
SRL: somatostatin receptor ligand
SSTR2: somatostatin type 2 receptor
